# Supplementary material for: The Interaction of Genotype and Environment Determines Variation in the Maize Kernel Ionome
Source: G3 (Bethesda). 2016 Oct 21;6(12):4175–83. doi: 10.1534/g3.116.034827 (PMC5144985; doi:10.1534/g3.116.034827)
Supplement: Supplemental Material [file supp_6_12_4175__index.html]

The Interaction of Genotype and Environment Determines Variation in the Maize Kernel Ionome — Supplemental Material 

# The Interaction of Genotype and Environment Determines Variation in the Maize Kernel Ionome

## Supplemental Material for Baxter *et al.*, 2016

**Files in this Data Supplement:**

- Table S1 - Growout Information. (.pdf, 761 KB)
- Table S2 - Percent Variance (R2) of Mo, Cd, and Ni QTL (.pdf, 395 KB)
- Table S3 - Location LOD Scores Compared to Seed Element Content (.pdf, 402 KB)
- Figure S1 - Heritability vs. Number of QTL (.tiff, 201 KB)
