# Supplementary material for: The Interaction of Genotype and Environment Determines Variation in the Maize Kernel Ionome
Source: G3 (Bethesda). 2016 Oct 21;6(12):4175–83. doi: 10.1534/g3.116.034827 (PMC5144985; doi:10.1534/g3.116.034827)
Supplement: Supplemental Material [file supp_g3.116.034827_TableS1.pdf]

Table S1. Growout Information

Location	Year	Planting Date	No. Lines	No. Line Reps.*	Genotyped Lines†	No. Lines Post-OR	Genotyped Lines Post-OR
Florida	2005	9/14/2005	220	1(118), 2(2)	176	180	147
Florida	2006	8/25/2006	118	1(71), 2(47)	95	114	94
Indiana	2009	5/9/2009	193	1	156	169	134
Indiana	2010	5/10/2010	168	1	139	155	129
North Carolina	2006	5/6/2006	197	1(19), 2(121), 3(53), 4(4)	160	187	151
New York	2005	5/9/2006	256	1(7), 2(50), 3(199)	209	249	204
New York	2006	5/9/2006	82	1(60), 2(22)	67	56	46
New York	2012	5/24/2012	168	1	137	128	104
Missouri	2006	5/17/2006	97	1(29), 2(50), 3(18)	81	58	50
South Africa	2010	11/2009	88	1	72	82	68

*No. lines with rep. in parentheses

†239 unique genotyped lines
