# Supplementary material for: The Interaction of Genotype and Environment Determines Variation in the Maize Kernel Ionome
Source: G3 (Bethesda). 2016 Oct 21;6(12):4175–83. doi: 10.1534/g3.116.034827 (PMC5144985; doi:10.1534/g3.116.034827)
Supplement: Supplemental Material [file supp_g3.116.034827_TableS2.pdf]

Table S2. Percent Variance (R^2) of Mo, Cd, and Ni QTL

	**Mo 1@378**	**Cd 2@215**	**Ni 9@7**
FL05	33.99	43.36	NA
FL06	27.13	27.08	NA
IN09	26.85	38.65	21.85
IN10	33.35	44.77	19.67
NC06	31.95	48.88	32.01
NY05	69.85	52.17	47.61
NY06	45.17	21.61	NA
NY12	57.19	60.44	35.10
MO06	58.21	NA	NA
SA10	NA	NA	NA

Percent variance for 3 QTL in locations where QTL is significant. QTL chromosome and position is indicated under element name.
