# Supplementary material for: The Interaction of Genotype and Environment Determines Variation in the Maize Kernel Ionome
Source: G3 (Bethesda). 2016 Oct 21;6(12):4175–83. doi: 10.1534/g3.116.034827 (PMC5144985; doi:10.1534/g3.116.034827)
Supplement: Supplemental Material [file supp_g3.116.034827_TableS3.pdf]

Table S3. Location LOD Scores Compared to Seed Element Content

	FL	IN	NY
Cd_2@214_LOD	16.96	23.81	37.13
Cd_2@214_normalizedLOD*	0.08	0.15	0.17
Avg_Cd	0.42	0.44	0.21
Mo_1@378_LOD	11.31	17.64	51.72
Mo_1@378_normalizedLOD*	0.06	0.11	0.24
Avg_Mo	3.22	4.85	1.99
Ni_9@7.7_LOD	0.47	8.12	23.25
Ni_9@7.7_normalizedLOD*	0.00	0.05	0.11
Avg_Ni	1.01	2.31	0.95

Comparison for top three significant QTL-by-location interaction loci (Cd, Mo, Ni)
*LOD scores normalized to population size
