# Supplementary figures and images for: The Interaction of Genotype and Environment Determines Variation in the Maize Kernel Ionome
Source: G3 (Bethesda). 2016 Oct 21;6(12):4175–83. doi: 10.1534/g3.116.034827 (PMC5144985; doi:10.1534/g3.116.034827)

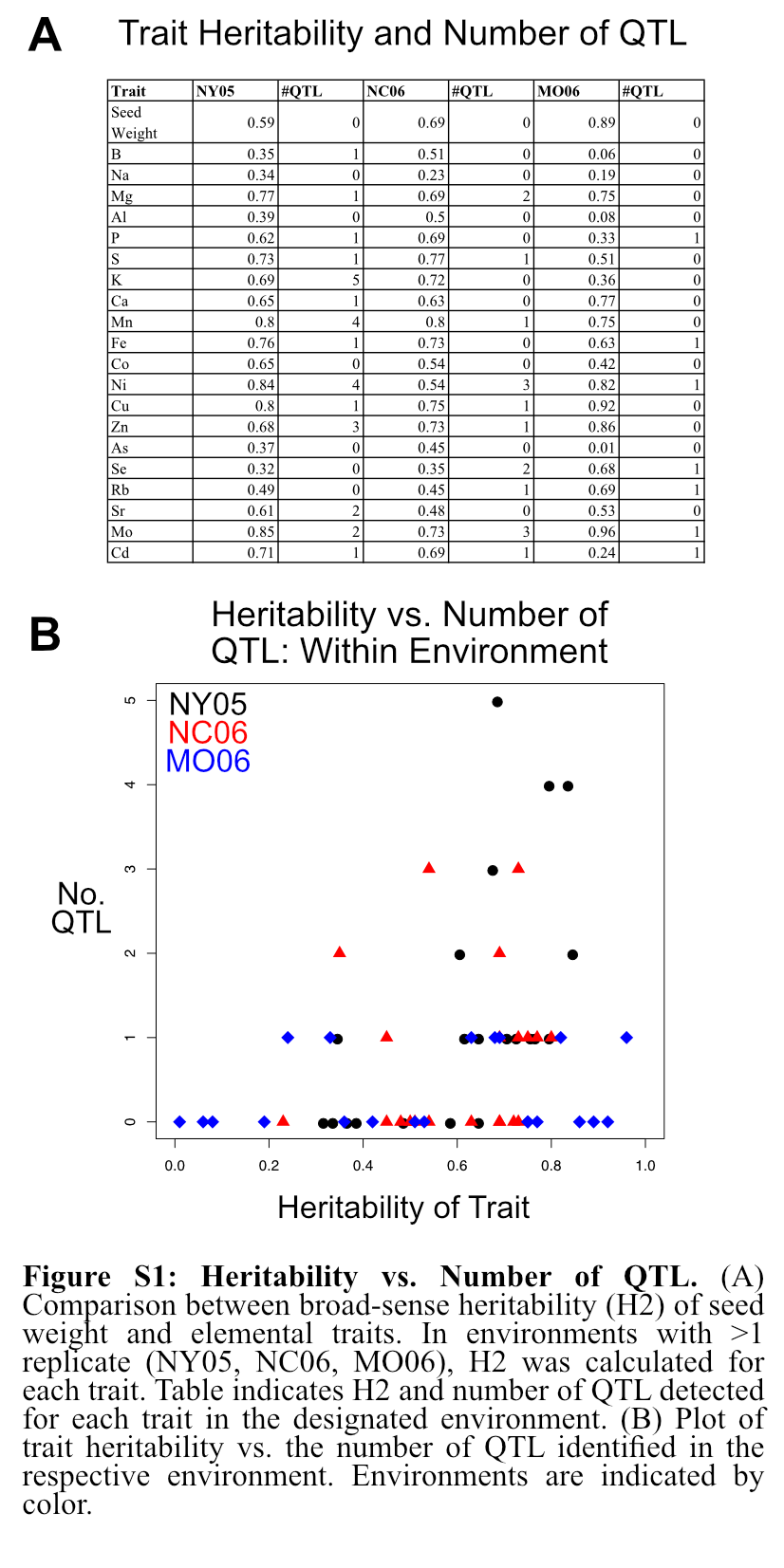

Supplement: Supplemental Material [file supp_g3.116.034827_FigureS1.tif]
